# Sintering Reaction and Pyrolysis Process Analysis of Al/Ta/PTFE

**DOI:** 10.3390/polym11091469

**Published:** 2019-09-09

**Authors:** Jun Zhang, Junyi Huang, Yuchun Li, Qiang Liu, Zhongshen Yu, Jiaxiang Wu, Zhenru Gao, Shuangzhang Wu, Jiaying Kui, Jiaxing Song

**Affiliations:** College of Field Engineering, PLA Army Engineering University, Nanjing 210007, China (J.Z.) (J.H.) (Z.Y.) (J.W.) (Z.G.) (S.W.) (J.K.) (J.S.)

**Keywords:** Al/Ta/PTFE, sintering reaction, TG-DSC, XRD, TaF_3_

## Abstract

When the Al/Ta/PTFE reactive material was sintered at 360 °C in a vacuum sintering furnace, it was found that the material reacted to form a soft fluffy white substance and carbon black. To explore the reaction process further, powder samples of pure PTFE, Al/PTFE, Ta/PTFE and Al/Ta/PTFE, and molded cylindrical specimens were prepared. A TG-DSC test was carried out on the thermal reaction of four reactive materials, and XRD phase analysis was conducted on the white product, formed by the sintering reaction and the residue of the TG-DSC test sample, based on which of the pyrolysis processes and reaction mechanisms were analyzed. The results show that Ta and PTFE could have a chemical reaction at sintering temperature (360 °C) to form soft and fluffy white material TaF_3_ and carbon black, which can overflow the surface of the specimen and cause cracking of the specimen, which is tightly pressed. Since no obvious exothermic peak showed up on the TG-DSC curve, the composition of the residue of TG-DSC sample at different temperatures was tested and TaF_3_ was detected in the residue at 350 °C and 360 °C, indicating that Ta began to react with PTFE at a temperature range of 340–350 °C. According to the chemical properties and product formation of Ta, it could be speculated that the reaction mechanism between Ta and PTFE involves the PTFE decomposing first, then the fluorine-containing gas product reacting with metal Ta. According to the temperature range of the reaction, it is estimated that PTFE starts to decompose before 500 °C, but it is not detected effectively by TG-DSC, and the introduction of Ta could also affect the decomposition process of PTFE.

## 1. Introduction

Metal fluoropolymer, generally referred to as a reactive material, is also known as impact-induced energetic material. It is a new type of advanced energetic material that can explode (deflagration) under high-speed impact and release a large amount of chemical energy, which makes it different from traditional explosives and propellant energetic material [1,2,3]. Since Willis et al. [4] found that Al/PTFE can react under high-speed impact conditions, it has become a hot spot in the research field of energetic materials. The material can be developed into new types of high-efficiency damaging warhead, increasing the capacities of anti-light armor and air defense. It can also be used as the propulsion system of space satellites or rockets. In the petroleum industry, it can also be used in perforating bombs to increase the efficiency of oil production [5].

Taking polytetrafluoroethylene (PTFE) as an example, whose chemical formula is –(CF_2_–CF_2_)–. PTFE, the chemical has the highest fluorine content in all fluoropolymers, up to 75% [6]. When it is decomposed by heat, PTFE will release highly oxidizing fluorine-containing radical, which could react intensely with various metals. This is because the bond energy of these metals with fluorine is much higher than the strength of C–F bond and is not lower than the metallic bond energy of any corresponding metal. Many scholars have conducted a lot of research on the key problems of PTFE-based metal reactive materials, including mechanical properties, preparation technology, reaction mechanisms, and energy release characteristics. Xu et al. [7] have thoroughly studied quasi-static mechanical properties, impact mechanical properties, and energy release reaction of Al/PTFE reactive materials. Nielson et al. [8,9,10] further enriched the formulation of metal polymers, summarized the commonly used active fillers, such as Al, Zr, Ti, Ni, and Ta., and improved the molding process of Al/PTFE reactive materials by replacing the “dry process” with “damp process” to make the mixing more uniform. The study also found that PTFE has different reaction mechanisms with different metals. Taking Al and Mg as examples, Al/PTFE is a mixed reaction mechanism [11], that is, PTFE decomposes first, and then its decomposition products react with Al. Koch. E. C et al. [12] monitored the reaction process by infrared spectroscopy and found that, in the condensed phase of Mg/PTFE, Mg first had Grignard reaction with molten PTFE to form a C–Mg–F structure, which disappeared with the temperature rising further, during which the product, MgF_2_, increased gradually. 

Although Al/PTFE reactive materials have higher strength and density than traditional energetic materials, there are still many problems to be solved in practical applications, such as inferior strength to metal components, insufficient penetration ability as a warhead; insufficient energy release level, as well as testing and research of constitutive relations of reactive materials. Some scholars have introduced metals (W, Ni) [13,14,15], metal oxides (Fe_2_O_3_, CuO_2_) [16,17], and metal hydrides (TiH_2_) [18] in Al/PTFE, with the aim of improving the mechanical properties or energy release rates of Al/PTFE reactive materials, on which much research has been conducted. Metal Ta is a kind of transition metal with high-density (16.65 g/cm^3^) [19] and high hardness (6.5) [20], which is much larger than that of Al (density is 2.7 g/cm^3^ and hardness is 2–2.9), and it has good heat transference, fracture toughness and corrosion resistance within a wide temperature range. Therefore, it is widely used in superalloys, high-temperature components, chemical equipment, and armor-piercing projectiles [21].

Due to its excellent physical and chemical properties, Ta was initially introduced into Al/PTFE reactive material as an additive in order to improve the mechanical properties of the material, and to explore its effects on the reaction characteristics and energy release levels of the reactive materials. During the preparation of vacuum sintering (sintering temperature is 360 °C), the reactive material could have a significant chemical reaction and produce a white product. No reports on this reaction phenomenon was found. To explore the reaction process and the causes, four groups of powdered materials and molded cylindrical specimens are prepared in this essay. The thermal decomposition and thermal reaction processes of these four groups of reactive materials are studied by TG-DSC, and the sintered product and the composition of the residue of TG-DSC test sample are tested by XRD. Finally, the reaction process and mechanism of the Al/Ta/PTFE system are analyzed based on the test results.

## 2. Experiment Section

### 2.1. Raw Material and Sample Preparation

Raw materials: PTFE (average particle size: 25 μm, density: 2.2 g·cm^−3^, from 3F, Shanghai, China); Al powder (average particle size: 6–7 μm, density: 2.7 g·cm^−3^, from JT-4, Hunan, China); Ta powder (average particle size: 45 μm, density: 16.68 g·cm^−3^, from Yinuo, Qinhuangdao, China). Uniform powder samples of pure PTFE, Al/PTFE, Ta/PTFE and Al/Ta/PTFE reactive materials were prepared, and the latter three materials were pressed into cylindrical specimens. Pure PTFE was used to explore the thermal decomposition process without additives. Al/PTFE and Ta/PTFE were used to eliminate the influence of Al on the reaction of Ta and PTFE.

The content of Al and PTFE in Al/PTFE and Al/Ta/PTFE materials was in accordance with the chemical equilibrium ratio (26.5 wt.%/73.5 wt.%). The mass fraction of metal Ta in Al/Ta/PTFE was set to 30% of the total mass. The amount of Al in Al/Ta/PTFE was replaced by Ta to obtain the content ratio of Ta/PTFE. The proportion of ingredients for each group of the reactive materials is shown in Table 1.

**Figure 1:**
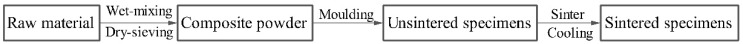
The typical preparation process of reactive materials.

**Figure 2:**
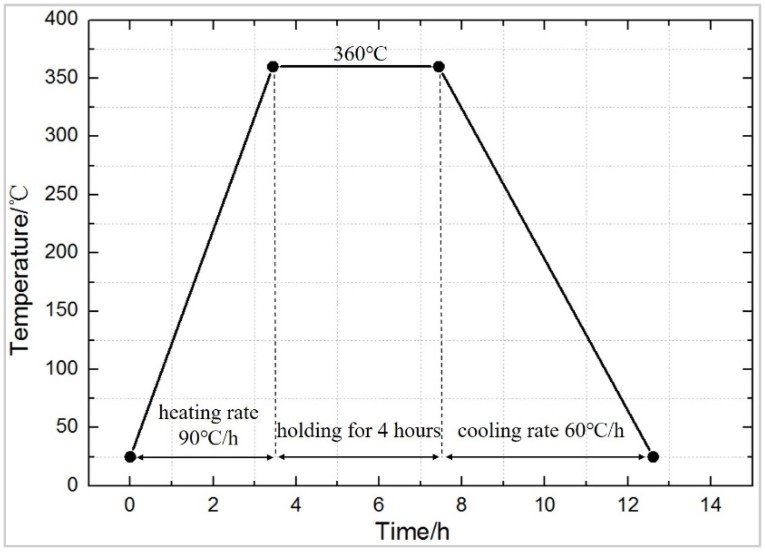
The typical sinter temperature curve of reactive materials.

The preparation process mainly includes mixing, molding, and sintering. The specific process is shown in Figure 1. The raw material is placed in a beaker, and appropriate amount of absolute ethanol is added and stirred by an electric mixer for 20 min, and then placed in a vacuum oven, dried at 60 °C until completely dried, and sieved (60 meshes) to obtain a uniform powder, which is pressed into cylindrical test specimens of a size Φ10 mm × 10 mm (pressing pressure was set at 240 MPa, holding time for 20 s), with a molding dye and FLS-30T hydraulic press (Rongmei, Taizhou, China). The pressed test specimen was placed in the air for 2 h to release the trapped air and the residual stress to avoid cracking, due to air expansion during sintering [22]. Then it was placed in a TL1200 vacuum (Boyuntong, Nanjing, China) sintering furnace, at a constant temperature of 360 °C for 4 h. It was set that the heating rate is 90 °C·h^−1^ and the cooling rate is 50 °C·h^−1^. Technological curve of the sintering temperature is shown in Figure 2.

### 2.2. Experimental Contents

The thermal decomposition and thermal reaction processes of four groups of the reactive materials were analyzed by German Bavaria Model NETZSCH-STA449C thermogravimetry-differential scanning calorimetry (TG-DSC, NETZSCH-STA449C, NETZSCH, Bavaria, Germany). The test temperature was 25–800 °C, and the heating rate at 10 °C/min. To prevent air from skewing the reaction, the experiment was carried out in a highly pure argon atmosphere, with the argon purge rate of 30 mL/min.

Moreover, a German Bruker D8 ADVANCE X-ray diffractometer (XRD, Bruker D8 ADVANCE, Bruker, Berlin, Germany) was used to detect the sintered product and the solid residue phase after thermal analysis, to analyze the composition of the product. The instrument parameters as set as follows: The tube voltage was 40 kV, the current was 40 mA, Cu K_α_ radiation (λ = 0.15406 nm), the scanning range 2θ was 10–90°, and the scanning speed was 5°·min^−1^.

## 3. Results and Discussion

### 3.1. Sintering Reaction of Ta/PTFE and Al/ Ta/PTFE and Analysis of the Sintering Products

**Figure 3:**
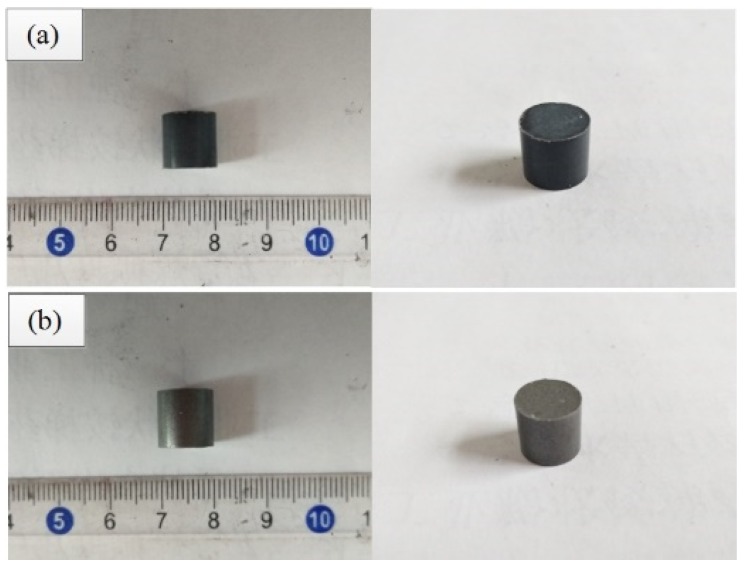
Specimens of two reactive materials before sintering (**a**) Ta/polytetrafluoroethylene (Ta/PTFE) specimen (**b**) Al/Ta/PTFE specimen.

**Figure 4:**
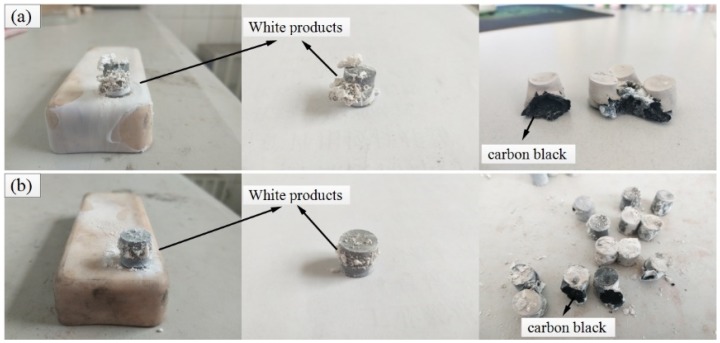
Specimens of two reactive materials after sintering (**a**) Ta/PTFE specimen (**b**) Al/Ta/PTFE specimen.

The cylindrical specimens, pressed by three types of reactive materials, are placed in a vacuum tube furnace, respectively, and sintered according to the temperature curve of the sintering process, as shown in Figure 2. The experiment demonsSintrated that Ta/PTFE and Al/Ta/PTFE chemically reacted and generated a large amount of soft and fluffy white powdery substances, attached to the outer surface of the test specimen, or scattered on the placement plate of the specimen. Some of specimens crack, showing carbon black inside. The state changes of specimens of these two reactive materials before, and after, sintering are shown in Figure 3, and Figure 4, respectively.

**Figure 5:**
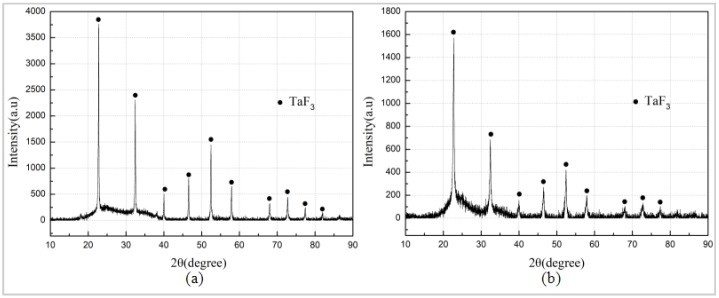
XRD (X-ray diffraction) results. (**a**) Sintered white products of Ta/PTFE (**b**) Sintered white products of Al/Ta/PTFE.

The Al/PTFE specimen did not react, and the specimen Ta/PTFE with more Ta content produced more white powder substances, from which it could be speculated that Ta and PTFE had chemical reaction under the sintering process. The white substances adhered to the surface of the two specimens, and the sample placement plate were subjected to XRD phase detection. The results are shown in Figure 5. It can be seen from the figures that the component of the white substances, produced by the two materials, is TaF_3_. However, no diffraction peak of AlF_3_ was found, indicating that Ta reacts with PTFE at this sintering temperature. The TaF_3_ may have a relatively small relative density lower than pressed specimens, resulting in an increase in the volume of the specimen, as well as expansion and cracking of the specimen. Moreover, its melting point is less than 360 °C, and it is easy to overflow the specimen and attach it to the surface or scatter on the placement plate of the test piece.

### 3.2. TG-DSC and XRD Phase Analysis of Four Groups of Reactive Materials

**Figure 6:**
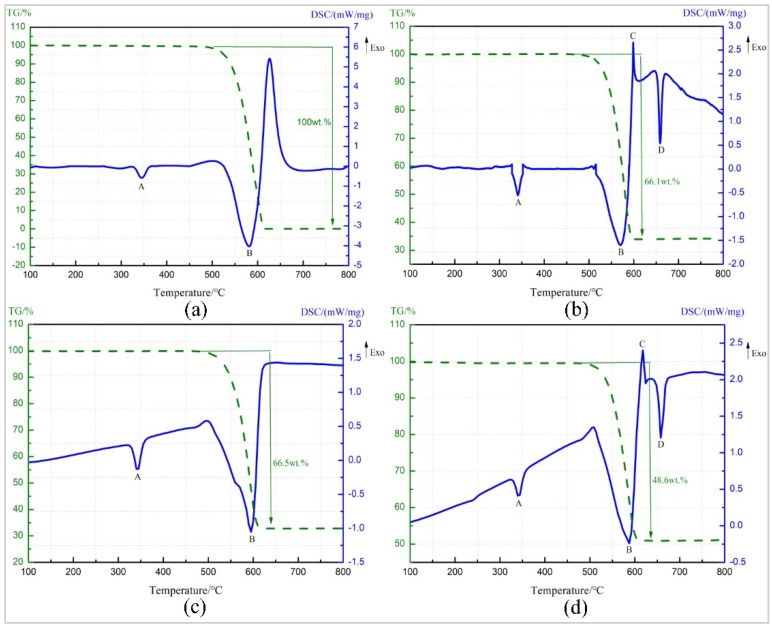
TG-DSC (Thermogravimetry-differential scanning calorimetry) curve of four materials. (**a**) Pure PTFE (**b**) Al/ PTFE (**c**) Ta/PTFE (**d**) Al/Ta/PTFE.

TG-DSC curve of pure PTFE is as shown in Figure 6a. The endothermic peak A starts at 325.2 °C and ends at 363.1 °C. The melting point of PTFE is 327 °C [23], and there is no change in the TG curve at this temperature stage. So peak A is the melting endothermic peak of PTFE. Starting from 509 °C, the TG curve shows a sharp drop in sample mass and the loss is 100%, and peak B starts at 514.02 °C, indicating that peak B is the endothermic decomposition peak of PTFE, and the decomposition product is gaseous product C_2_F_4_ with the boiling point of −76 °C [24].

Figure 6b shows TG-DSC curve of Al/PTFE. The endothermic peaks, A and B, were caused by the melting and decomposition of PTFE, which correspond to the TG-DSC test results of pure PTFE. Exothermic peak C starts at 597.9 °C, resulting from exothermic reaction between Al powder and PTFE decomposition product. The products are AlF_3_ and C (carbon) [25,26]. The boiling point of the produced AlF_3_ is low and could not hinder the reaction by covering on the surface of Al particles [27]. The peak temperature of peak D is 659 °C, resulting from heat absorption of Al powder, which has not reacted completely at about 660 °C [24].

The TG-DSC curve recorded for the Ta/PTFE is depicted in Figure 6c. The temperature range, corresponding to endothermic peak A is 331.0–353.5 °C, within which the sample mass remained unchanged, so peak A was also caused by fusion of PTFE. Peak B starts at 475.8 °C and ends at 615.9 °C and is the endothermic decomposition peak of PTFE. Within this temperature scope, TG curve shows the sample mass drops 66.5%, which is lower than the content ratio 70%. It is speculated that the reason is that decomposed PTFE reacts partially with Ta and could not overflow in the form of gaseous product. It is worth noting that the curve does not show a significant reaction exothermic peak around 360 °C.

Figure 6d presents the TG-DSC curve for Al/Ta/PTFE. Peak A and peak B are also the melting endothermic peak and decomposition peaks of PTFE, respectively. It can be seen from the TG-DSC curve of Al/PTFE that peak C is the reaction exothermic peak of Al and PTFE, and peak D is the melting endothermic peak of Al at about 660 °C. The contents of Al and PTFE in the specimen is a chemical equilibrium ratio. PTFE reacts with Ta before 360 °C, resulting in excessive Al, so the melting endothermic peak of Al powder appears. However, no significant exothermic peak appears on TG-DSC curve at around 360 °C.

The information about peak A of the four materials is shown in Table 2. It can be seen from the data in the table that metal additives can slightly delay the starting temperature of the endothermic peak, and the effect of the metal Ta is more obvious. However, they had little effect on the peak temperature. Pure PTFE has the highest melting enthalpy. The materials containing the metal additive reduced the content of PTFE and caused the value of melting enthalpy to decrease. In addition, the reaction of metal Ta with PTFE also reduced the content of PTFE, and the reaction exotherm further reduced the melting enthalpy of Ta/PTFE and Al/Ta/PTFE.

After TG-DSC test, XRD phase analysis was conducted on the specimen residue at 330, 340, 350, and 360 °C, respectively. The results show that TaF_3_ exists only in the sample residue at the temperature of 350 °C and above, indicating that Ta and PTFE begin to react within 340–350 °C, and produce TaF_3_. Since the reaction between Ta and PTFE coincides with the melting process of PTFE, and possibly that less heat is released during the reaction, TG-DSC curve does not show significant exothermic peak.

### 3.3. Analysis of Reaction Mechanism of Al/Ta/PTFE System

At certain temperatures, PTFE can react intensely with metals, such as Al, Mg, and Ti, but studies have shown that there are different reaction mechanisms. Take reactions with Al, Mg, and PTFE as examples. Before the pre-ignition reaction, Mg can have Grignard reaction with the molten PTFE to form a fluorine-based Grignard reagent. When heated to 500 °C, a C–Mg–F structure is found to exist in the Mg/PTFE system. When the temperature exceeds 700 °C, the structure disappears gradually, and the product is MgF_2_ [12]. However, the reaction between Al and PTFE is a mixed reaction mechanism, that is, PTFE decomposes first, and then its decomposition products react with Al [11].

Stable tantalum pentoxide (Ta_2_O_5_) forming on the surface of Ta could make it highly resistant to corrosion. However, the oxide film on the surface is destroyed at high temperatures, and easily oxidized, so it can react with various oxidizing substances. When the temperature is lower than 150 °C, it can react with fluorine, hydrofluoric acid, fluoride-containing acidic solution, and sulfur trioxide. From 200 °C, it starts to oxidize slightly and is oxidized obviously at 280 °C. When PTFE is decomposed by heat, it will release fluorine-containing radicals with strong oxidizing property. According to the chemical properties of Ta and PTFE, and the composition of its products, it could be speculated that the reaction mechanism of Ta and PTFE is similar to that of Al/PTFE. PTFE decomposes first, and the produced fluorine-containing ions have redox reaction with Ta, whose protective film has been destroyed to produce TaF_3_ and carbon black. Based on the above analysis results, the possible reaction involved is shown by Formulas (1) and (2):

(1) $${\left(C_{2}F_{4}\right)}_{n}\to nC_{2}F_{4}$$

(2) $$C_{2}F_{4}+\mathrm{Ta}\to {\mathrm{TaF}}_{3}+C$$

The TG-DSC curves of pure PTFE and Al/PTFE in Section 3.2 show that PTFE starts to decompose from about 500 °C. It also starts to react at 340–350 °C when metal Ta is added, which does not conform to the reaction mechanism of Ta/PTFE system obviously. The major reasons are as follows: First, numerous studies have shown that PTFE begins to decompose before 500 °C, for example, studies conducted by C. M. Simon and W. Kaminsky showed that PTFE started to decompose weakly at 260 °C and decomposes obviously when the temperature exceeds 400 °C [28]. Arai N also found that PTFE decomposed thermally at 350–400 °C and produced gaseous products [29]. When studying the influences of Al particle size on the pyrolysis reaction of Al/PTFE, Osborne and Pantoya pointed out that at 400 °C, PTFE could separate the F ion to have pre-ignition reaction with Al_2_O_3_ on the surface of the nano-Al [25]. The temperature at which Ta reacts with PTFE agrees well with the decomposition temperature range of PTFE, proposed in the literature mentioned above, which cannot be reflected on the curve in the TG-DSC test of pure PTFE and Al/PTFE because the initial thermal decomposition of PTFE is weak. Second, related thermogram studies have shown that metal powder has a great influence on the direction of the fluoropolymer structural process and the pyrolysis rate. The activation energy of the fluoropolymer with metal is about 1/2 to 1/3 lower than that of the pure polymer [11]. The introduction of metal Ta may affect the decomposition of PTFE, resulting in subsequent reactions. However, more experiments are required to fully understand the PTFE reactions.

## 4. Conclusions

After the sintering reaction of Al/Ta/PTFE was discovered, powder samples and cylindrical specimens for sintering the reactive materials of pure PTFE, Al/PTFE, Ta/PTFE, and Al/Ta/PTFE were prepared in this essay. TG-DSC test is adopted to analyze the thermal reaction process of the four groups of reactive materials, and the XRD phase is used to test sintered product and the residue of the TG-DSC reaction samples. By analyzing the pyrolysis process and the reaction mechanism, the results are obtained as follows:

1. Ta and PTFE chemically react when heated to 360 °C to form a soft and fluffy white material TaF_3_ and carbon black. TaF_3_ can overflow the surface of the specimen, causing cracking of the compacted cylindrical specimen, and scatter on the placement plate of specimens. This is presumably because the density of the substance is lower than that of the pressed specimens and the melting point is less than 360 °C.
2. The results of the XRD phase detection show that there is TaF_3_ in the residue of TG-DSC specimen at 350 °C and 360 °C, indicating that Ta and PTFE have reacted at 340–350 °C. However, no obvious reaction exothermic peak is found on TG-DSC curves of Ta/PTFE and Al/Ta/PTFE. It is possibly because the reaction generates little energy and coincides with the melting process of PTFE, and the liberated heat is absorbed for PTFE to melt.
3. It is speculated that the reaction mechanism of the Ta/PTFE system is that PTFE decomposes first, and then the product reacts with the highly oxidizable metal Ta to generate TaF_3_ and carbon black. TG-DSC test of PTFE and Al/PTFE shows that the decomposition temperature of PTFE starts at about 500 °C, which does not agree with the scope of the reaction temperature. It is speculated that PTFE does decompose before 500 °C, which could not be detected effectively, because the decomposition is weak, or the introduction of the metal Ta could affect decomposition of PTFE. Therefore, Ta reacts with PTFE during the sintering process.

## Figures and Tables

**Table 1:** Component ratios of four groups of reactive materials.

Reactive Material	Composition (wt.%)		
	Al	Ta	PTFE
PTFE	-	-	100
Al/PTFE	26.5	-	73.5
Ta/PTFE	-	48.55	51.45
Al/Ta/PTFE	18.55	30	51.45

**Table 2:** The information on peak A of four materials.

Materials	Starting Temperature/°C	Peak Temperature/°C	Termination Temperature/°C	Melting Enthalpy/J·g^−1^
PTFE	325.2	344.45	363.1	78.56
Al/PTFE	329.8	341.4	350.6	52.54
Ta/PTFE	331.0	343.0	353.5	29.57
Al/Ta/PTFE	330.5	342.4	353.1	40.37
